# Kinetic of the Maternally‐Derived Anti‐Parvovirus IgG During the Neonatal Period: A Quantitative Assessment

**DOI:** 10.1002/vms3.70429

**Published:** 2025-06-10

**Authors:** Yalda Tamaddon, Mehran Bakhshesh, Farnoush Arfaee, Mohammad Hossein Fallah Mehrabadi, Nader Azadi

**Affiliations:** ^1^ Department of Clinical Sciences, SR. C., Islamic Azad University, Tehran, Iran Islamic Azad University Tehran Iran; ^2^ Department of Animal Virology, Razi Vaccine and Serum Research Institute Research and Diagnosis, Agricultural Research, Education and Organization (AREEO), Karaj, Iran; ^3^ Departement of Poultry Diseases, Razi Vaccine and Serum Research Institute Research and Diagnosis, Agricultural Research, Education and Organization (AREEO), Karaj, Iran; ^4^ Veterinary Clinic Tehran Iran

**Keywords:** individual variations, parvovirus protection, passive IgG, persistence

## Abstract

**Background:**

Passive immunity (mainly IgG) protects canine neonates from the highly pathogenic and contagious canine parvovirus, while it can also largely interfere with parvovirus early vaccinations.

**Objectives:**

Quantitative evaluation of the anti‐parvovirus IgG transferred to newborn puppies from immunised bitches during the neonatal period, as the influence of individual variations on the kinetics of the specific IgG was also estimated.

**Methods:**

Sera samples from 80 new born puppies were taken at 10 day intervals in a dog kennel and quantitatively assessed for specific anti‐parvovirus IgG with a commercial ELISA kit. The resultant data were subjected to specific statistical analysis.

**Results:**

Statistical analysis definitely showed that the anti‐parvovirus IgG sufficiently transferred from the vaccinated bitches to their offspring, so that the average titre calculated was 5675.5 and 2625.9 in the 10 and 20 day old puppies, respectively, which were both considered significantly higher than the protective threshold titre (810). In our setting, however, the average anti‐parvovirus IgG titre of the one‐month old puppies was not significantly estimated protective, 959.7 (median = 15.57) with a coefficient of variation of 148.6%. It was also estimated that factors including bitches’ age, litters’ diversity, puppies’ age, the period from parvovirus vaccination to parturition and litter size reduced the passive anti‐parvovirus IgG levels in the newborn puppies, while bitches’ parity evaluated as a positive factor to boost passive anti‐parvovirus IgG titre.

**Conclusions:**

These results raise our knowledge on the kinetics of passive anti‐parvovirus IgG in newborn puppies and are useful for establishing parvovirus vaccination schedules.

## Graphical abstract

Anti‐parvovirus IgG sufficiently transferred from the vaccinated bitches to their offspring and remains above the protective threshold titre for one month after puppies’ birth.

Passive anti‐parvovirus IgG is affected by individual variations in that bitches’ parity evaluated as a positive factor to boost passive anti‐parvovirus IgG titre.

Further studies with large sample sizes that also include bitches’ sera will result in more comprehensive and accurate data about the transfer and persistence of the specific IgG.

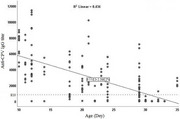

## 1 Introduction

Transfer of the maternal antibodies to carnivore neonates is always dependent on colostrum ingestion, particularly during the first hours of their lives and newborn's survival is entirely dependent on the adequate intake of colostrum and the window of susceptibility. As the newborn's passive immunity declines during early life, the infants become susceptible to infectious diseases. Therefore, it is critical that the mothers maintain sufficient immunity during pregnancy and, then, adequately transfer this pivotal immunity to their offspring after birth. However, avoiding interference from maternal antibodies is a key factor for the successful immunisation of newborn puppies (Pollock and Carmichael 1982; Chappuis 1998; Pereira et al. 2019; Mazzaferro 2020).

Immunoglobulin G is the main immunologic factor which is transferred from bitches to puppies and efficiently protects puppies from systemic and local infections during early life (Chastant and Mila 2019). It has been well documented that maternally derived antibodies (MDA) and more specifically the serum IgG concentration in the newborn puppies is significantly correlated with their health and fitting growth (Mila and Feugier et al. 2014; Mila and Grellet et al. 2014). It is, therefore, critically important that newborn puppies receive a sufficient amount of IgG, particularly during the first hours after birth (Chastant‐Maillard et al. 2017; Mila et al. 2018). Both genetic and environmental factors contribute to the proper transfer of maternal IgG to the neonate. This transfer, however, can vary between bitches and their offspring, impacting the intake of adequate IgG levels (Mila et al. 2015; Chastant‐Maillard et al. 2017; Chastant and Mila 2019).

Canine parvovirus (CPV) is one of the most lethal agents of young puppies. The etiologic virus known as CPV‐2 has been identified since the 1970s, spread rapidly worldwide and has continuously evolved to create several types and variants (Kelly 1978). Vaccination is the principal prophylaxis procedure for CPV, however due to multiple complicated factors, CPV vaccination failure is not uncommon (Altman et al. 2017). Interfering of the MDA with vaccination through neutralisation with modified live vaccines (MDV) is considered to be the most important reason for CPV immunisation failure and it is strongly recommended that the final puppy immunisation is administered when the MDA has sufficiently declined (Pollock and Carmichael 1982; Waner et al. 1996; Decaro et al. 2020). Despite inconsistencies reported (Elia et al. 2005; Cavalli et al. 2008; Cavalli et al. 2021) and its practical limitations, haemagglutination inhibition (HI) has been known as the gold standard test for evaluation of dog immunity against CPV, in which the HI titre of ≥ 1/80 is believed to be the protective titre against CPV (Buonavoglia et al. 1992; Pollock and Carmichael 1982; Carmichael et al. 1983; Decaro et al. 2005; Cavalli et al. 2008; Paul et al. 2006; Day et al. 2016). As the vital role of passive immunoglobulin G in puppies’ immunity against the deadly parvovirus, it is of interest to assess the kinetics of the specific anti‐parvovirus IgG in the newborn puppies. For this purpose, we quantitatively measured the amount of the passively transferred anti‐parvovirus IgG as well as its duration in Belgian Malinois newborn puppies from immune bitches in a dog kennel. We also statistically evaluated the possible influence of individual variations on the uptake and persistence of this specific IgG during the neonatal period.

## 2 Materials and Methods

### 2.1 Study Population

Eighty newborn puppies from 12 Belgian Shepherd (*Belgian Malinois*) bitches, which were born during 2019 to 2021 in a dog kennel located in Alborz Province, Iran, were included in this study. Detailed information of the bitches and puppies which were included in the study is presented in Table 1. The bitches were between 2 and 7 years with an average of 3.85 years and an average litter size of 6.15. The bitches were fully vaccinated against CPV‐2 using the VANGUARD plus 5 L4 (Zeotis, USA) vaccine and the mean period of vaccination to parturition was 14.77 months. The kennel was in good health and management condition: all dogs were provided standard housing facilities, diet and veterinary care.

**TABLE 1:** Detailed information about the bitches and their puppies which were subjected to this study. The bitches were labelled A‐L and their age, litter size, parity number and the period from CPV vaccination to parturition are given in the table.

Bitches’ information					Puppies’ information	
Bitches	Age (Year)	Litter size (%)	Parity number	Period from parovirus vaccination to parturition (Month)	Puppies’ sex Number (%)	Puppies’ first Sampled age (day) Number (%)
A	2	7 (8.7)	1	8	Male 43 (53.7)	∼ Day 10 48 (60)
B	4	4 (5)	2	22		
C	7	3 (3.7)	5	12		
D	2	8 (10)	1	15		
E	5	8 (10)	3	29		∼ Day 20 19 (23.75)
^*^E(R)	4	9 (11.5)	2	12		
F	4	8 (10)	4	12	Female 37 (46.2)	
G	3	5 (6.2)	1	8		
H	2	6 (7.5)	1	16		∼ Day 30 13 (16.25)
I	5	6 (7.5)	1	11		
J	3	3 (3.7)	2	15		
K	4	7 (8.7)	1	5		
L	5	6 (7.5)	2	27		

* Bitch E gave birth twice during the study, named E and *E(R). The number and percentage of puppies sampled at each stage were described. Puppies’ information including their sex and their number (%) at the first sampling were also explained. Of the total 80 puppies, 39 (48.75%), 19 (23.75%) and 13 (16.25%) were sampled 3, 2 and 1 times, respectively. Puppies borne from the bitch E (R) which comprised 9 (11.5%) of the total puppies were only sampled one time (day 10).

Using clot activator tubes, blood samples were taken from newborn puppies and we were not allowed to take samples from the bitches. With slight variations, the samples were taken at the age of 10, 20 and 30 days after birth (Table 1). After centrifugation at 3000 RPM for 5 min, the serum samples were separated and kept on ‐20°C until tested.

### 2.2 Quantitative ELISA for Anti‐CPV IgG

Specific anti‐parvovirus IgG in the puppies’ sera was quantitatively measured by using the commercial CPV IgG ELISA test kit (Demeditec, Kiel, Germany) according to the manufacturer's instructions. Briefly, conserved CPV antigen capable of detecting IgG antibodies against all CPV strains is coated to the 96‐well‐microtitre plate and incubated for 75 min at 37°C. The plate was washed and diluted with negative/positive controls as well as sera samples were added to the wells and incubated for 60 min at 37°C. Puppies’ sera were thawed and diluted 1:150 in the specific buffer provided in the kit. Serial dilutions of both the negative control (three 3‐fold serial dilutions of 1:100 to 1:900) and positive controls (five 3‐fold serial dilutions of 1:100 to 1:8100) were also applied. The plate was washed, 100 µL of conjugated anti‐species antibody added to each well and incubated for 60 min at 37°C. After washing the plate, 100 µL of the mixed substrate was dispensed to each well and incubated for 10 min in the dark at room temperature. Finally, 50 µL of stop solution was added to each well.

The optical density (OD) was measured at 450 nm using 620 nm as a reference on the ELISA reader. The final anti‐parvovirus IgG titre of each sample was calculated by constructing a curve in EXCEL software using a cut‐off line (five 3‐fold serial dilutions of positive control 1:100 to 1:8100). According to the kit's manual, the IgG antibody threshold titre of 810 is considered as protective against parvovirus. The manufacturer states that the sensitivity and specificity of the kit are 98.3% and > 96%, respectively, compared to the HI and the threshold protection titre was determined with both the HI test and challenge with the wild type virus (personal communications).

### 2.3 Statistical Analysis

Quantitative variables are expressed as mean and the standard deviation. Also, qualitative variables including frequency and percentage of puppies in each litter were calculated (Table 1). In the univariate analysis, variables with a *p*‐value of less than 0.2 were included in the multivariable regression model analysis, as the variable with a p‐value of less than 0.05 considered to be significant. Linear mixed model (LMM) was used to estimate the decline of the maternal anti‐parvovirus IgG antibody during the neonatal period on days 10, 20 and 30, which were entered into the model as independent variables.

Using two‐way analysis of variance (ANOVA), the relationship between quantitative variables and passive anti‐parvovirus IgG titre in newborn puppies was evaluated. These variables included litters' anti‐parvovirus IgG mean titre, bitches' age and variation, bitches' parity, puppies' age, the period from parvovirus vaccination to parturition, puppies' sex and litter size.

Qualitative ordinal variables were calculated using a Chi‐square (χ^2^) test. The influence of sex on the intake and duration of the maternal anti‐parvovirus IgG titre in the newborn puppies was assessed by using an independent T test. We also used a Kaplan‐Meier plot to predict the survival of the anti‐parvovirus IgG titre during the neonatal period at 10 days after birth, as the *p*‐value of less than 0.05 was considered statistically significant.

Using a Cox proportional‐hazard regression model, hazard ratios (HRs) were estimated with a 95% confidence interval (CI). A Cox multivariable model was used to control the effect of confounding variables and also to predict the relationship between the variables. If the cofounder or interactions between the variables was observed, it was reported separately. The proportional hazard (PH) assumption was assessed based on the scaled Schoenfeld residual plot. All statistical tests were conducted based on the predicted hypothesis and no adjustment was applied for the multiple analyses as the p‐values ˂ 0.05 were considered significant. All statistical analyses were performed by using R software version 4.0.3.

## 3 Results

### 3.1 Passive Anti‐Parvovirus IgG Obtained by the Neonates and Its Persistence

**GRAPH 1:**
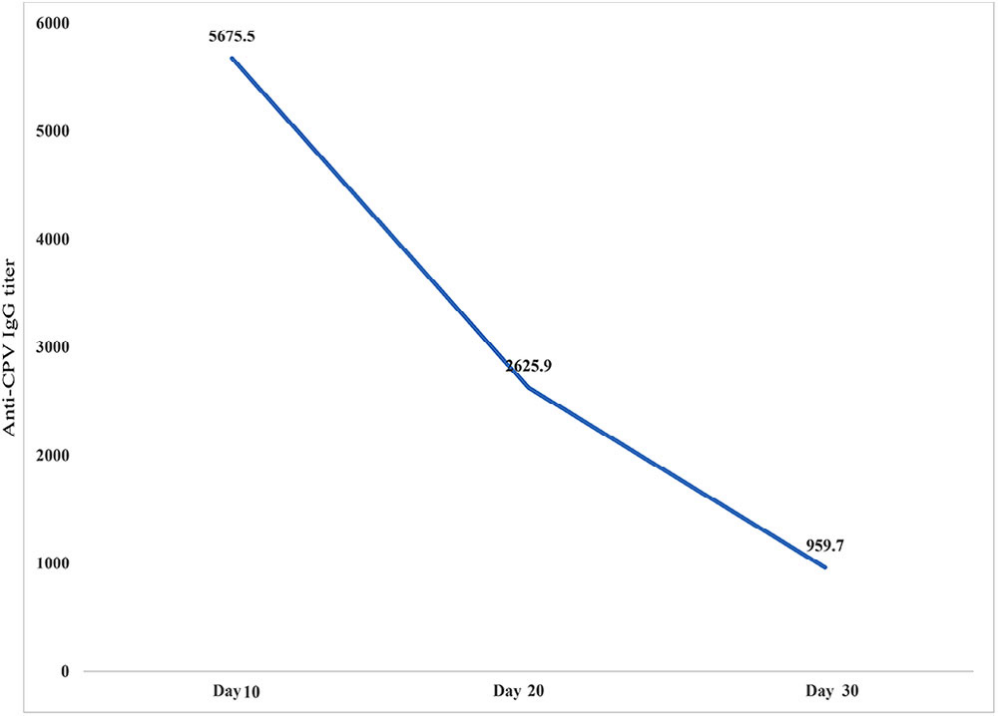
The average decline of the anti‐parvovirus IgG titre during the first month of the puppies’ life in the kennel. The means of the anti‐parvovirus IgG titre in puppies at the ages of 10, 20 and 30 days, are represented.

Our analyses showed that the newborn puppies in the kennel obtained a sufficient level of specific anti‐parvovirus IgG from their bitches. The average titre of the specific anti‐parvovirus IgG in 10 day old puppies is estimated as 5675.5 (median = 4872.5), with the coefficient of variation (CV) of 51%. The average titre decreased to 2625.9 (median = 2154.9) and a higher CV of 87.5 % in 20 day old puppies. The average titre of the 10 and 20 day old puppies were both significantly higher than the protective threshold titre of 810 (*p < 0.001*). At around one month of age, the average of anti‐parvovirus IgG titre in the puppies greatly reduced to 959.7 (median = 15.57) with a CV of 148.6%, which was not found to be significantly higher than the protective threshold titre (p < 0.42) (Graph 1 and Table 2). At this age, 57.5% of the puppies were also below the protective threshold titre of anti‐parvovirus IgG. These data suggest that the puppies in the setting of our study likely possess insufficient anti‐parvovirus IgG titre at one month of age.

**TABLE 2:** Number of puppies which were sampled at each step is depicted. Means, medians, standard deviations (SD), coefficient of variations (CV) and statistical differences between the mean of the anti‐parvovirus IgG titre of the puppies at each age of sampling with the protective threshold (810) are exhibited.

Age (Day)	N	Mean	SD	Median	CV	*p*‐value (Re = 810)
10 day	48	5675.5	2778.9	4872.5	51 %	**< 0.001**
20 day	59	2625.9	2299.2	2154.9	87.5 %	**< 0.001**
30 day	69	959.7	1549.2	15.57	148.6 %	**0.42**

**GRAPH 2:**
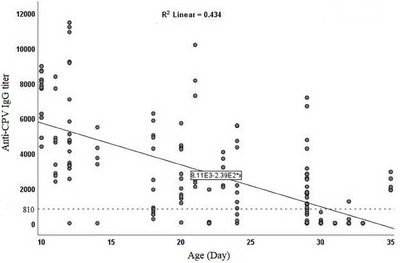
The linear regression graph predicted that the passive anti‐parvovirus IgG decline is correlated with the puppies’ age (*R^2^ = 0.434*) and also estimated that the titre totally decayed by the age of 40 days in the kennel. The dotted line represents the protective threshold titre (810) and the black dots are individual puppies sampled at each stage.

**GRAPH 3:**
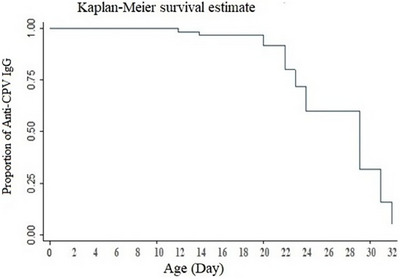
Kaplan‐Meier plot showing the survival rate of the passive anti‐parvovirus IgG in the newborn puppies during the first month of life. The plot estimated that on days 23, 29 and 31, the puppies had lost an average of 25%, 50% and 75% of their anti‐parvovirus IgG titre, respectively.

The linear regression graph also predicted a linear correlation between the passive anti‐parvovirus IgG decay and puppies’ age (*R^2^ = 0.434*) estimating that the titre would not have been detectable by the age of 40 days in the kennel (Graph 2). The average reduction of the specific IgG from days 10 to 20 and days 20 to 30 were both significant (*p < 0.001*) and estimated at 1812.7 and 2833.2, respectively, implying that the specific passive IgG declined faster as the age increased (Table 3). Consistently, a Kaplan‐Meier plot also predicted that at days 23, 29 and 31, puppies had lost an average of 25%, 50% and 75% of their specific IgG titre, respectively, estimating that it would not be traceable 35 days after the puppies’ birth (Graph 3).

**TABLE 3:** The average declines of the anti‐parvovirus IgG titre at 10 day intervals compared to the titre at day 10 as the reference titre which were both significant (*p* < 0.001). The average reduction of the titre from day 10 to 20 and day 20 to 30 was estimated at 1812.7 and 2833.2, respectively.

Age (day)	Mean (SD)	95 % CI	*P* value
10 day	*Re* 5675.5	—	—
20 day	−1812.7 (1549.2)	−2733 to ‐ 892.2	**< 0.001**
30 day	−2833.2 (2299.2)	−3800.5 to ‐1866.0	**< 0.001**

### 3.2 Factors Influencing Passive Anti‐Parvovirus IgG Levels in the Newborn Puppies

**GRAPH 4:**
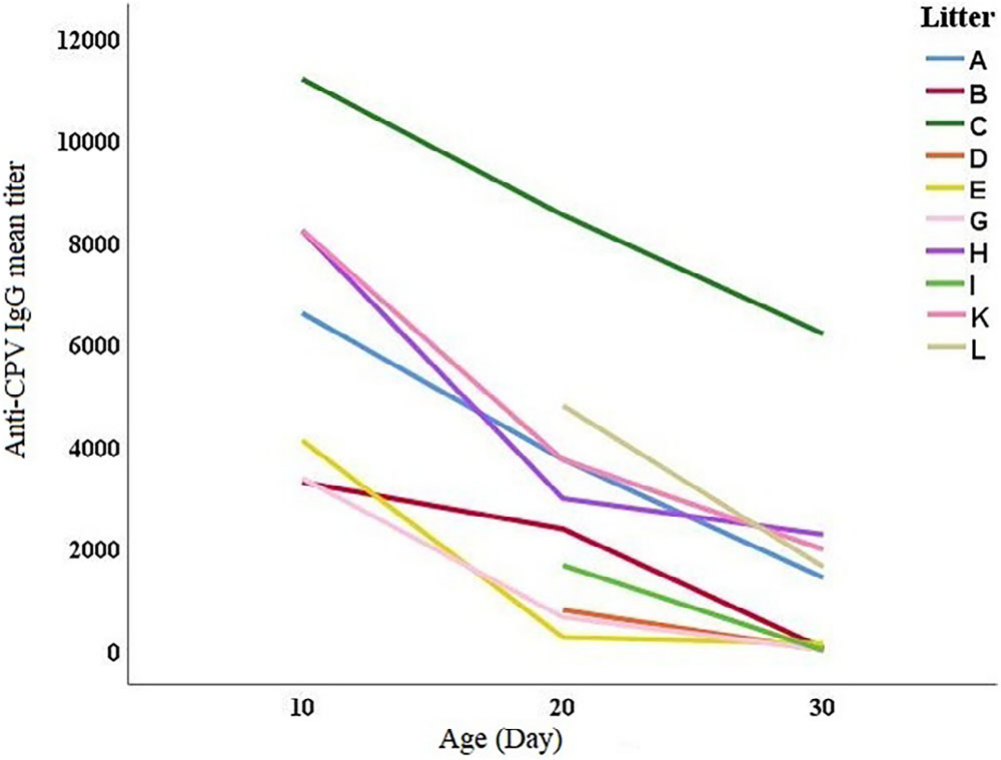
The average amount of the passive anti‐parvovirus IgG titre in the newborn puppies of each individual litter on days 10, 20 and 30 after birth. The newborn puppies from each individual litter exhibited a significantly different average of anti‐parvovirus IgG titre at three steps of sampling and exhibited a unique trend of titre decline during the first month of life. Litters D, I and L were only available for sampling at the second and third steps. Litter E(R) was available for sampling only at the first step and litters F and J were only available at the 3^rd^ sampling and therefore, they were not included in the graph.

The influence of factors including bitches’ age, puppies’ age, bitches’ parity, bitches’ variation, puppies’ sex, period from parvovirus vaccination to parturition and litter size on passive anti‐parvovirus IgG levels in the newborn puppies was investigated. Using multivariable regression analysis, it was determined that bitches’ parity number may increase passive anti‐parvovirus IgG levels in the newborn puppies (Coefficient = 1488.024, *p value* = 0). However, bitches’ age, puppies’ age, litter diversity, the period from parvovirus vaccination to parturition and litter size were estimated to reduce passive anti‐parvovirus IgG and its persistence in the newborn puppies (Table 4). Graph 4 shows the average amount of anti‐parvovirus IgG titre in the newborn puppies of each individual litter at day 10, 20 and 30 after birth. The newborn puppies from each of the individual litters in our study possessed a significantly different average of anti‐parvovirus IgG titre at all 3 steps of sampling (*p value* = 0.000) and exhibited a unique trend of titre decline during the first month of life.

**TABLE 4:** The influence of factors including bitches’ age, puppies’ age, bitches’ parity, bitches’ variation, puppies’ sex, period from parvovirus vaccination to parturition and litter size on passive anti‐parvovirus IgG titre in newborn puppies, evaluated by multivariable regression analysis. The values of the impacts were signified as coefficient with the *P‐* value ˂ 0.05. Except forparity number, all factors appeared to significantly reduce passive anti‐parvovirus IgG levels in the newborn puppies.

Risk factors	Coefficient	SE	*P* value	95% CI	
**Puppies’ age**	−229.026	17.69101	**0**	−263.7	−194.353
**Puppies’ sex**	−262.552	285.4396	**0.358**	−822.004	296.8992
**Bitches’ variation**	−241	57.69856	**0**	−354.088	−127.913
**Parity**	1488.024	278.8171	**0**	941.5524	2034.496
**Bitches’ age**	−476.087	187.4697	**0.011**	−843.521	−108.653
**Period from parovirus vaccination to parturition**	−167.625	22.83519	**0**	−212.381	−122.869
**Litter size**	−466.212	107.4807	**0**	−676.87	−255.554

While female puppies exhibited an insignificantly higher (*p value* = 0.473) amount of anti‐parvovirus IgG titre (5968.78) than male's (5406.6) on day 10 after birth, their average titre decreased to the lower amount (883.7) than the male's (1014) at the age of one month. The difference between the female and male puppies’ anti‐parvovirus IgG titre was also not statistically significant in multivariable regression analysis (*p value* = 0.358) (Table 4).

## 4 Discussion

Passively transferred immunoglobulin G (IgG) is vital for newborn puppies to stay in good health and grow. It is, therefore, critical that maternal IgG adequately transfer to the offspring with sufficient performance to protect the newborn puppies during their early life (Chastant‐Maillard et al. 2017; Chastant and Mila 2019; Pereira et al. 2019). Research has also shown that individual and environmental variations can influence the transfer and persistence of maternal IgG to canine neonates (Mila et al. 2015; Chastant‐Maillard et al. 2017; Chastant and Mila 2019). However, interference of the maternally transferred IgG as a major immunogenic factor of colostrum with parvovirus vaccination is also a major concern in dog immunisation programs (Goddard and Leisewitz 2010; Mazzaferro 2020). Transfer and decline of total IgG levels has previously been investigated in newborn puppies and the serum concentration of IgG was less than the threshold of 2.3 g/L at day 2, which was determined to be deficient making the neonates at increased risk of mortality and lower growth rate (Mila and Grellet et al., 2014; Mila et al. 2018). With respect to the maternally derived CPV type ‐2 specific antibody titre (CPV2 MDA) and the total serum concentration of IgG in the canine neonates, a study found a positive correlation between CPV2 MDA and total serum concentration of IgG at day 2 but not at day 28 (Mila et al. 2018). A recent study also found a discrepancy between the HI and ELISA tests to detect MDA's against CPV in neonates (Dall'Ara et al. 2021).

Our data definitely show that the newborn puppies from vaccinated Belgian Malinois bitches possessed high levels of passive anti‐parvovirus IgG on day 10 after birth. However, the average titre significantly reduced at 10 days intervals until day 30, when the mean and median of the specific passive anti‐parvovirus IgG titre in 80 puppies was calculated as 959.7 and 15.57 respectively, with a very high CV of 148.6%, which was not consistent with a protective titre. Our Kaplan‐Meier plot also predicted that passive anti‐parvovirus IgG reduced more rapidly in puppies after the age of 20 days. In line with these calculations, our linear regression model also estimated that the specific passive titre would not have been detectable by the age of 40 days in the kennel. The decay of passive anti‐parvovirus IgG measured by the ELISA test in our study exhibited a different pattern from the one previously reported for anti‐CPV MDA evaluated by the HI test (Pollock and Carmichael 1982; Gooding and Robinson 1982; Waner et al. 1996). Based on the HI testing, these studies determined a half‐life of 9.7 to 11.6 ± 2.5 days for anti‐CPV MDA decay, while our assessments for the survival of the passive anti‐parvovirus IgG show that its decay drastically sped up with age, which is not comparable to the half‐life defined by the HI test. Future studies will determine the reason(s) for this difference in which the possible role of individual and environmental factors need to be carefully defined. Management and, particularly, density of the dogs in the colonies are crucial risk factors that can potentially increase the viral load neutralising puppies’ passive immunities, and are highly recommended to be considered in future research. Although the role of risk factors including breed size, litter size and growth rate in the duration of the specific anti‐CPV MDA has been documented (Goddard and Leisewitz 2010; Mila and Grellet et al., 2014), future studies are required to more clearly define the kinetics of passively transferred IgG to newborn puppies in association with individual and environmental variations. In addition, the degree of protectiveness of maternally derived anti‐parvovirus IgG (measured by ELISA test) and its correlation with specific passively transferred immunity against parvovirus also needs to be comprehensively investigated and compared to the conventional HI test.

The role of individual and environmental variations on the transfer of specific anti‐IgG from dams to offspring has been illustrated (Mila and Grellet et al., 2014; Mila et al. 2015; Chastant‐Maillard et al. 2017; Chastant and Mila 2019). As a novel finding, we report that the bitches’ parity number may enhance passive IgG levels in their neonates. A report from Dall’ Ara et al. (2015) also found a significant positive correlation between parity and the concentration of IgG in the foetal amniotic fluid. These results, however, suggest that increasing parity can boost the concentration of passively transferred IgG and probably the quality of colostrum are also reported in the goat, cow and sow (Connelly et al. 2013; Buranakarl et al. 2021; Chniter et al. 2016; Nuntapaitoon et al. 2019; Segura et al. 2020; Grodkowska et al. 2023).

Our statistical analysis also revealed that increasing the bitches’ age, puppies’ age, litters diversity, period from parvovirus vaccination to parturition and litter size negatively influenced the specific maternal IgG in newborn puppies. The intra litter variation of the passive IgG serum concentration has also been previously reported (Chastant‐Maillard et al. 2017; Chastant and Mila 2019). We also found significantly different averages for the anti‐parvovirus IgG titre between litters at all three stages of samplings. However, in comparison to the data from Mila and Grellet (2014), we found a significantly negative impact of litter size on maternal IgG in newborn puppies. The insignificant difference which was estimated between the passive anti‐CPV IgG average titres in the female and male puppies, consistent with the previous report (Mila and Grellet et al., 2014), suggest that sex has no significant effect on receiving maternal IgG by canine neonates.

As a portion of the total passive IgG, this quantitatively study also determined that maternal anti‐parvovirus IgG can be considered as a marker for the kinetic assessment of total maternal IgG during the canine neonatal period. These results, with the influence of individual and environmental variations described on the passive anti‐parvovirus IgG during the neonatal period are useful information for scientists in the field, veterinarians and dog owners and are also valuable for improving parvovirus prophylactic programmes. However, to achieve more comprehensive data, it is of interest to design studies that comprise more settings and also to analyse and compare the anti‐parvovirus IgG in both bitches and their newborn puppies together. It is also recommended to determine the minimum level of the passive anti‐parvovirus IgG that interferes with parvovirus MDV, proposing optimal timing of vaccination.

## 5 Conclusion

In our study newborn puppies sufficiently obtained passive anti‐parvovirus IgG via MDA, however levels declined below the threshold of protection one month after birth. Passive anti‐parvovirus IgG is influenced by individual and environmental factors during the neonatal period, in which the possible positive effect of the parity number is a novel significant finding.

## Author Contributions

**Yalda Tamadon**: investigation. **Mehran Bakhshesh**: conceptualisation, supervision, funding acquisition, validation, methodology, project administration, visualisation, resources, writing – original draft, writing – review and editing. **Farnoush Arfaee**: project administration. **Mohammad Hossein Fallah**: formal analysis, data curation. **Nader Azadi**: methodology, funding acquisition.

## Ethics Statement

Dogs care and the experimental steps were performed in full accordance with the criteria of care and use of institutional animals Medical Sciences Ethics Committee of Islamic Azad University of Science and Research Branch, Tehran, Iran (Ethical code: IR.IAU.SRB.REC.1399.051).

## Data Availability

The data that support the findings of this study are available from the corresponding author upon reasonable request. The data are not publicly available due to privacy or ethical restrictions.
